# Soft Tissue Graft Placement Using a Porcine Acellular Dermal Matrix (PADM) and Resorbable Magnesium Fixation Screws: A Case Series

**DOI:** 10.3390/medicina61071144

**Published:** 2025-06-25

**Authors:** Giorgio Tabanella, Patrick Rider, Svenja Rogge, Kristina Tseneva, Ivana Butorac Prpić, Željka Perić Kačarević

**Affiliations:** 1 O.R.E.C.—Oral Reconstruction and Education Center, Via Rovereto 6, 00198 Rome, Italy; gtabanella@gmail.com; 2 Botiss Biomaterials AG, Ullsteinstrasse 108, 12109 Berlin, Germany; patrick.rider@botiss.com (P.R.); svenja.rogge@botiss.com (S.R.); kristina.tseneva@botiss.com (K.T.); 3 Department of Dental Medicine, Faculty of Dental Medicine and Health Osijek, J.J. Strossmayer University of Osijek, Crkvena 21, 31 000 Osijek, Croatia; butoracivana88@gmail.com; 4 Department of Anatomy, Histology, Embriology, Pathology Anatomy and Pathology Histology, Faculty of Dental Medicine and Health Osijek, J.J. Strossmayer University of Osijek, Crkvena 21, 31 000 Osijek, Croatia

**Keywords:** soft tissue graft, resorbable magnesium, fixation screw

## Abstract

*Background and Objectives*: Adequate soft tissue thickness and keratinized mucosa are essential for the long-term health and esthetics of the peri-implant area. A porcine acellular dermal matrix (PADM) has shown promise in augmenting soft tissue, but reliable fixation remains a challenge. *Materials and Methods*: This case series describes the use of a PADM fixed with resorbable magnesium screws (NOVAMag^®^) in three patients requiring peri-implant soft tissue augmentation. The grafts were stabilized with magnesium screws on the buccal side. The clinical outcomes were evaluated over a period of 3–6 months using STL imaging and direct measurements. *Results*: All patients showed an improvement in their mucosal volume and keratinization. The mean vertical increase in soft tissue was 0.87 ± 0.16 mm and the mean horizontal increase was 1.00 ± 0.13 mm. The mucosal thickness increased from a baseline value of 1.0–1.2 mm to 1.9–2.1 mm, and the width of the keratinized mucosa improved by an average of 1.0 mm. No complications were observed, and in all cases there was tension-free healing and esthetic results. *Conclusions*: A PADM in combination with resorbable magnesium fixation screws offers a predictable and minimally invasive solution to improve peri-implant soft tissue with favourable short-term volumetric and esthetic results.

## 1. Introduction

The importance and practice of soft tissue augmentation has become more prominent over the past half a century [1], as the requirements for assessing the success of oral implant therapy have become increasingly stringent [2]. For patient satisfaction, it is required that the peri-implant soft tissue looks as natural as possible. This is in addition to the classic evaluation of success in implant therapy in terms of implant survival, the stability of the prosthetic work over a long period of time, the absence of infection and adequate osseointegration [2,3,4]. Therefore, the evaluated success is a combination of the functional restoration and the subjective satisfaction of the patient, which relies heavily on the esthetic outcome [5].

It has been established that a mucosal thickness of 2 mm or less impairs the peri-implant soft tissue stability and that the presence of less than 2 mm of keratinized mucosa affects the long-term soft tissue stability [6,7]. This has led to an increasing interest in soft tissue augmentation techniques. The main clinical situations associated with soft tissue grafts can be divided into recession coverage, keratinized tissue augmentation and soft tissue volume augmentation [8]. Despite the numerous surgical techniques available and biomaterials used, autogenous connective tissue grafts (CTGs) and free gingival grafts (FGGs) are still considered the gold standard. Despite the biological advantages of autogenous tissue grafts, harvesting tissue from the palatal donor site prolongs the surgical time and is dependent on the anatomical characteristics of the donor site, which means there is a limited availability and patient-dependent quality of the grafted tissue. These procedures are also associated with high patient morbidity, manifested as severe postoperative discomfort, pain and bleeding at the donor site [9,10]. Furthermore, in terms of healing and esthetics, FGGs have been reported to result in poor colour matching to the surrounding tissue [11,12].

To overcome these shortcomings, the focus has shifted to connective tissue substitutes. In contrast to autogenous transplants, these materials of xenogeneic, allogeneic or synthetic origin reduce the morbidity, shorten the operation time and offer high availability. Porcine acellular dermal matrices (PADMs) are recognized as a replacement for the gold standard in periodontal and implant surgery [13,14,15,16,17,18]. They consist of collagen types I and III and elastin without additional artificial cross-linking [19,20]. According to the literature, PADMs can promote the growth and proliferation of human gingival fibroblasts, osteoblasts and endothelial cells, resulting in strong revascularization in the early healing phase [15,20,21]. Lin et al. [22] also showed that a PADM promotes the migration, adhesion and proliferation of periodontal ligament cells and human oral fibroblasts. As the PADM is repopulated by the cells of the surrounding soft tissue, the final colour of the regenerated soft tissue matches that of the surrounding tissues for a better esthetic look.

There are various options for securing soft tissue grafts, such as sutures, screws and pins. The standard for securing soft tissue grafts is via sutures; however this does have some disadvantages in comparison to using screws. Handling the soft tissue graft and retaining its exact position in the intended place when using sutures requires additional attention and planning during surgery, whereas the use of a screw immediately secures the soft tissue graft in the intended position and in direct contact with the wound bed, without micromovements, which enables the easy integration of the graft with the native tissue. Additionally, the ability of screws to compress the soft tissue graft to ensure close contact with the wound bed can have positive consequences for the overall volume of soft tissue gained.

In this case series, we present the use of resorbable metal fixation screws for securing soft tissue grafts. The fixation of soft tissue grafts using titanium screws has previously been reported [23]; however this has the clear disadvantage that the screws need to be removed after the regenerative period. The use of resorbable polymeric screws has already been shown as a viable alternative to titanium screws for the fixation of soft tissue grafts for applications such as the reconstruction of the anterior cruciate ligament [24]. However, the use of polymeric screws has been related to issues such as a slow resorption time [25], allergic reactions [26] and foreign body reactions that can even occur up to 12 months post implantation [27].

Since the 20th century, magnesium has been successfully used as a biomaterial in various areas of medicine, such as orthopedics and vascular and general surgery [28,29,30,31]. More recently it has been applied in dentistry for bone regeneration [32,33,34]. A resorbable metal fixation screw is made of a magnesium alloy and has previously been demonstrated as an efficient means for securing barrier membranes [35]. The screw degrades within one year [36], transforming into non-toxic byproducts [37] that are completely resorbed [35], such as magnesium ions, which are naturally present within the human body [36].

The aim of this paper is to present a new technique for soft tissue augmentation using a PADM and a magnesium fixation system based on a series of cases. Although porcine acellular dermal matrices (PADMs) have been used previously for soft tissue augmentation, a novel aspect of our approach is the use of resorbable magnesium fixation screws to stabilize the PADMs during healing. To our knowledge, this is the first clinical case series reporting the use of magnesium-based fixation for the placement of PADMs in soft tissue augmentation.

## 2. Materials and Methods

### 2.1. Surgical Protocol

All the clinical procedures in this case series were performed between January and December 2024 by one of the co-authors (G.T.) in a dental practice in Rome, Italy (the clinical procedures were reviewed and approved by the Comitato Etico Territoriale Lazio Area 2; approval code: 51.23PUd CET2 aslrm2). The following inclusion and exclusion criteria were used to select the cases.

The inclusion criteria were patients who required soft tissue augmentation in previously augmented or implant-supported sites; the presence of inadequately keratinized mucosa or a thin soft tissue biotype; good general health; and a non-smoker or light smoker status (≤10 cigarettes/day).

The exclusion criteria included uncontrolled systemic diseases (e.g., diabetes), poor oral hygiene, active periodontal or peri-implant infections, the use of anticoagulants or immunosuppressive drugs and heavy smoking.

**Figure 1:**
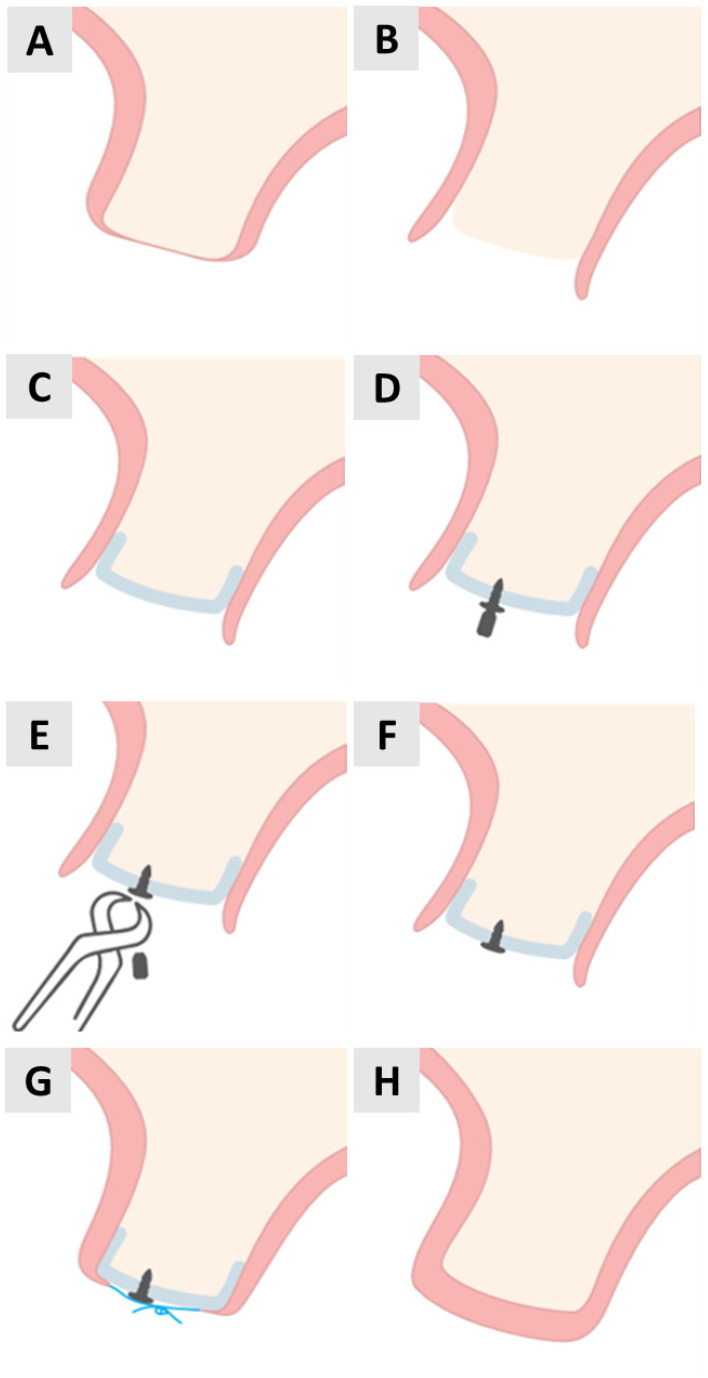
Step-by-step illustration of PADM placement and fixation with magnesium screw for soft tissue augmentation. (**A**) Initial anatomy before surgery. (**B**) Crestal incision. (**C**) Flap elevation. (**D**) Positioning PADM and drilling. (**E**) Screw insertion begins. (**F**) Screw fully seated. (**G**) Suturing. (**H**) Postoperative closure.

Before we present the clinical situations, we would like to show schematically how this innovative surgical technique was performed. The soft tissue and bone are shown preoperatively (Figure 1A). A local infiltration of articaine (4% articaine with 1:100,000 adrenaline; Septodont, Saint-Maur-des-Fossés, France) was administered. A crestal incision was made to access the site (Figure 1B), taking special care to preserve the keratinized tissue.

Using a microelevator (Tabanella 2, Hu-Friedy Mfg. Co., LLC, Chicago, IL, USA) followed by a wider elevator (Prichard), the full-thickness flap was carefully elevated (Figure 1C).

Prior to placement, the PADM (mucoderm^®^, botiss biomaterials GmbH, Zossen, Germany) was hydrated in saline for 2 min. It was then cut to size, adjusted to the implanted area and positioned under the flap. While it was stabilized with tissue pliers, a pilot hole was drilled through the PADM and into the patient’s native bone in preparation for magnesium screw placement (Figure 1D). The fixation screws were placed on the buccal aspect of the alveolar ridge to ensure the stable positioning of the PADM.

The magnesium screws (NOVAMag^®^ Fixation Screws S-XL, botiss biomaterials GmbH, Zossen, Germany) were inserted through the PADM until the flat screw head exerted uniform pressure on the wound bed (Figure 1E,F). Periosteal incisions were then made to obtain a coronally advanced flap that could completely cover the PADM. The flap was secured using horizontally reinforced mattress sutures using non-absorbable synthetic 5.0 monofilament sutures (Figure 1G). The procedure ended with a completely closed wound and the complete stabilization of the PADM under the soft tissue (Figure 1H).

### 2.2. Oral Scanning

The augmentation site was scanned using an intraoral scanner (iTero Elements Plus Series^®^, Align Technology, San Jose, CA, USA) prior to the augmentation surgery and then 3 months after surgery. The preoperative and postoperative STL files were superimposed on each other and aligned using reproducible anatomical points. The software then automatically calculated regions with a changing volume, which were indicated via colour representation in the superimposed scans (Table 1).

## 3. Results

### 3.1. Case 1

**Figure 2:**
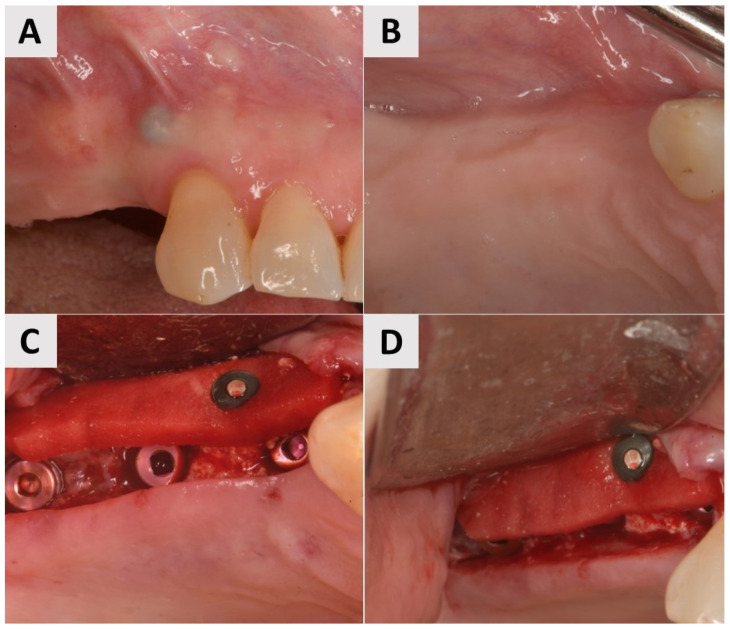
Clinical phases of surgical procedure for Case 1. (**A**,**B**) Soft tissue before second stage of surgery. Bucco-lingual collapse of ridge, as well as insufficient band of keratinized mucosa, is evident. (**C**,**D**) Surgical site after insertion of PADM soft tissue graft, stabilized with 7 mm magnesium screw.

A 69-year-old female patient presented with an obvious bucco-lingual collapse of the alveolar ridge and an insufficient band of keratinized mucosa (Figure 2A,B). Six months after bone augmentation using a xenograft (cerabone^®^ plus, botiss biomaterials GmbH, Zossen, Germany), three implants (Nobel Biocare^®^, Nobel Biocare AG, Kloten, Switzerland) were inserted. The augmentation of the soft tissue was performed in a second step to improve the mucosal conditions and stabilize both the implants and the surrounding tissue. A flap was elevated and a PADM graft (mucoderm^®^, botiss biomaterials GmbH, Zossen, Germany) was inserted and stabilized with a 7 mm magnesium screw (NOVAMag^®^ fixation screw, size S, botiss biomaterials GmbH, Zossen, Germany) to improve the adaptation to the recipient site and achieve greater stability compared to that offered by conventional periosteal sutures (Figure 2C,D). The surgical site was closed using reinforced 5.0 e-PTFE horizontal mattress sutures.

**Figure 3:**
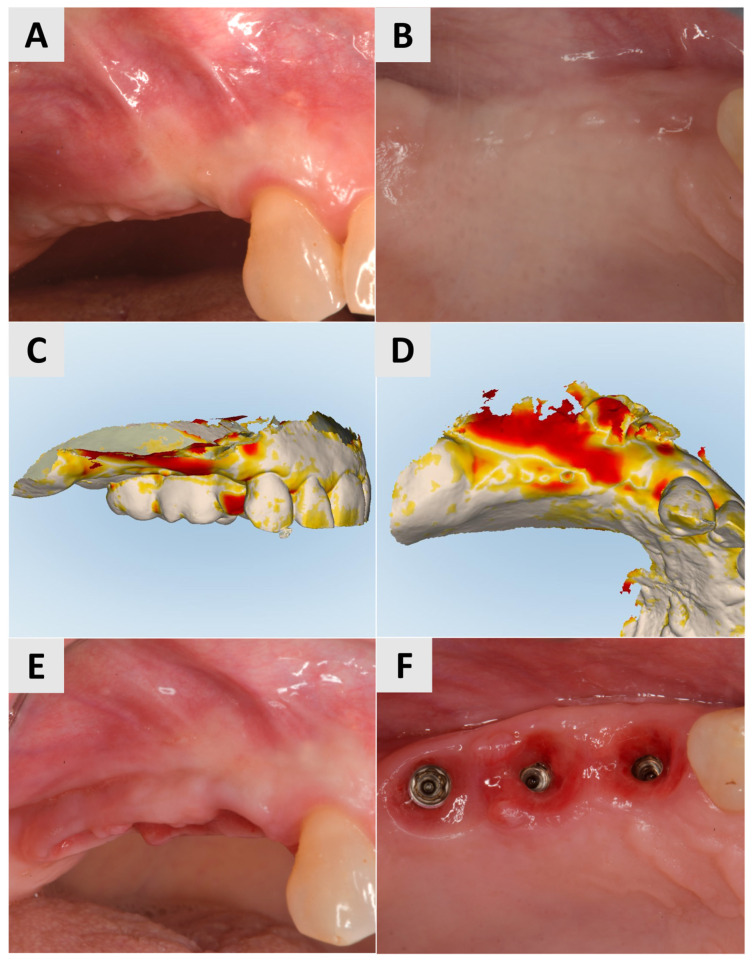
Follow-up for Case 1 at 3-7 months after surgery. (**A**,**B**) 3 months postop after mucogingival plastic surgery. Improved mucosal keratinization and thickness as well as vestibular depth evident. (**C**,**D**) STL imaging 3 months after surgery, showing successful soft tissue augmentation. (**C**) Vertical soft tissue gain of >1 mm. (**D**) Horizontal soft tissue gain of >1 mm. (**E**,**F**) Implant site 7 months postop, ready for crown placement. Suitable gingival contour and mucosal thickness evident.

Three months after surgery, a larger band of keratinized mucosa and a deeper vestibule as well as the improved thickness and profile of the buccal mucosa were noted. STL imaging confirmed an increase in soft tissue of more than 1 mm in both the vertical and horizontal dimensions (Figure 3C,D). After 7 months, the patient returned for crown restoration. The results showed a thickened band of keratinized mucosa and adequate ridge height, resulting in a satisfactory esthetic and functional outcome (Figure 3E,F). The final result showed a significant improvement in the soft tissue architecture and stability of the implant site compared to the initial state (Figure 3A,B).

### 3.2. Case 2

**Figure 4:**
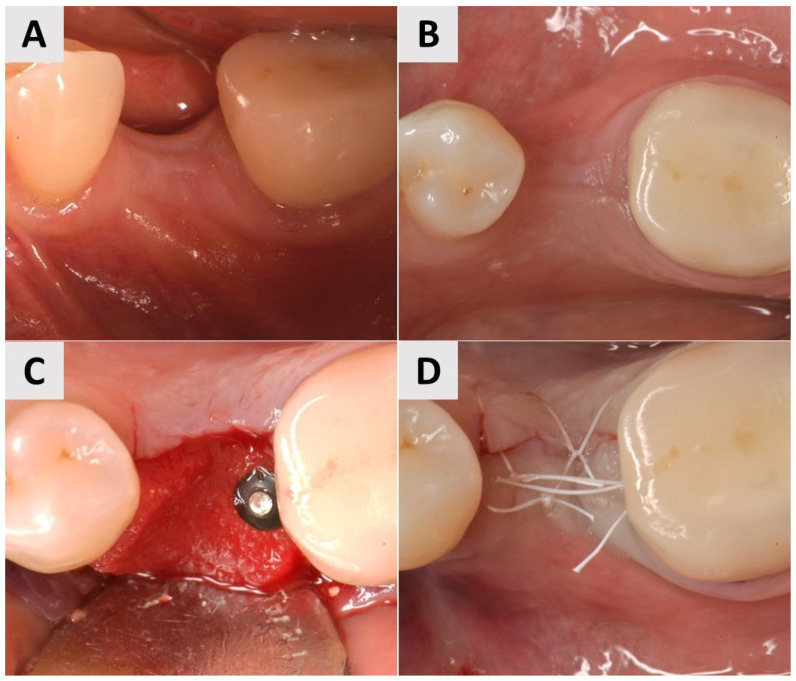
Clinical phases of surgical procedure for Case 2. (**A**,**B**) Soft tissue profile before surgical intervention. Narrow band of keratinized mucosa and buccal concavity evident. (**C**) Surgical site after insertion of PADM graft and fixation with 7 mm magnesium screw. (**D**) Surgical site after wound closure using reinforced 5.0 e-PTFE horizontal mattress sutures.

A female patient, 40 years old, showed a narrow band of keratinized mucosa and a clear buccal concavity (Figure 4A,B). To improve both their mucogingival profile and volume, a full-thickness flap was elevated and then a PADM graft (mucoderm^®^, botiss biomaterials GmbH, Zossen, Germany) was inserted. The graft was stabilized using a 7 mm magnesium fixation screw (NOVAMag^®^ fixation screw, size S, botiss biomaterials GmbH, Zossen, Germany) to ensure secure adaptation to the recipient site (Figure 4C). To facilitate primary wound healing, the surgical site was closed with reinforced horizontal 5.0 e-PTFE mattress sutures, achieving tension-free closure and the complete coverage of the graft (Figure 4D).

**Figure 5:**
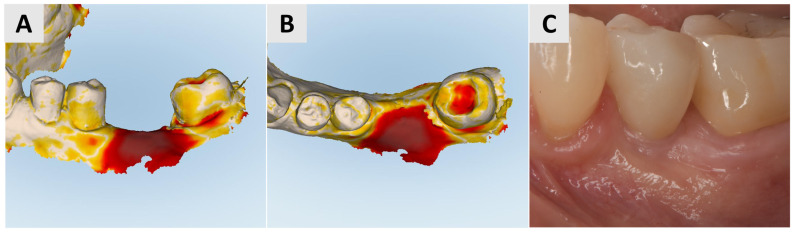
Follow-up for Case 2 at 3-5 months after surgery. (**A**,**B**) STL imaging 3 months after surgery, showing successful soft tissue augmentation. (**A**) Vertical soft tissue gain of >0.75 mm. (**B**) Horizontal soft tissue gain of >0.75 mm. (**C**) 5 months postop after crown placement.

At the 3-month follow-up, STL imaging showed an increase in soft tissue of more than 0.75 mm in both the vertical and horizontal directions, confirming successful augmentation (Figure 5A,B). Five months after surgery, the soft tissue profile had visibly improved, and the keratinization and mucosal thickness had increased. A definitive crown was inserted and completed the rehabilitation with an esthetically and functionally satisfactory result (Figure 5C).

### 3.3. Case 3

**Figure 6:**
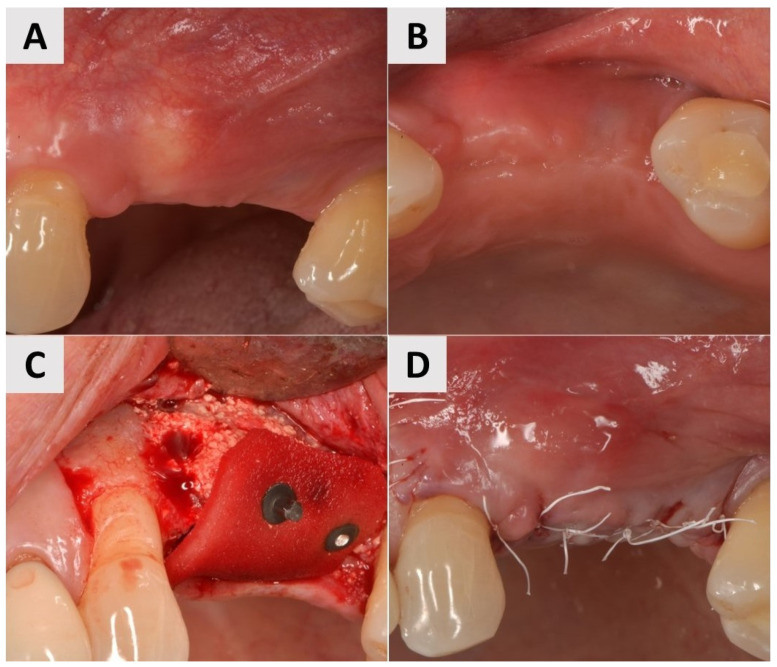
Clinical phases of surgical soft tissue augmentation for Case 3. (**A**,**B**) Soft tissue before surgical intervention. Lack of keratinization and sufficient thickness of mucosa visible. (**C**) Surgical site after implant placement and fixation of PADM graft with 7 mm magnesium fixation screws. (**D**) Surgical site closure using reinforced horizontal mattress sutures.

A 73-year-old male patient presented with insufficient keratinization and an insufficient mucosal thickness. In a second surgical procedure performed 6 months after bone augmentation with a xenograft (cerabone^®^ plus, botiss biomaterials GmbH, Zossen, Germany), two implants (Nobel Biocare^®^, Nobel Biocare AG, Kloten, Switzerland) were inserted. To improve the volume and contour of the soft tissue, a PADM graft (mucoderm^®^, botiss biomaterials GmbH, Zossen, Germany) was inserted and fixed with 7 mm magnesium fixation screws (NOVAMag^®^ fixation screws, size S, botiss biomaterials GmbH, Zossen, Germany) to promote vertical and horizontal tissue augmentation (Figure 6A–C). The site was then closed with reinforced horizontal 5.0 e-PTFE mattress sutures, allowing for tension-free healing (Figure 6D).

**Figure 7:**
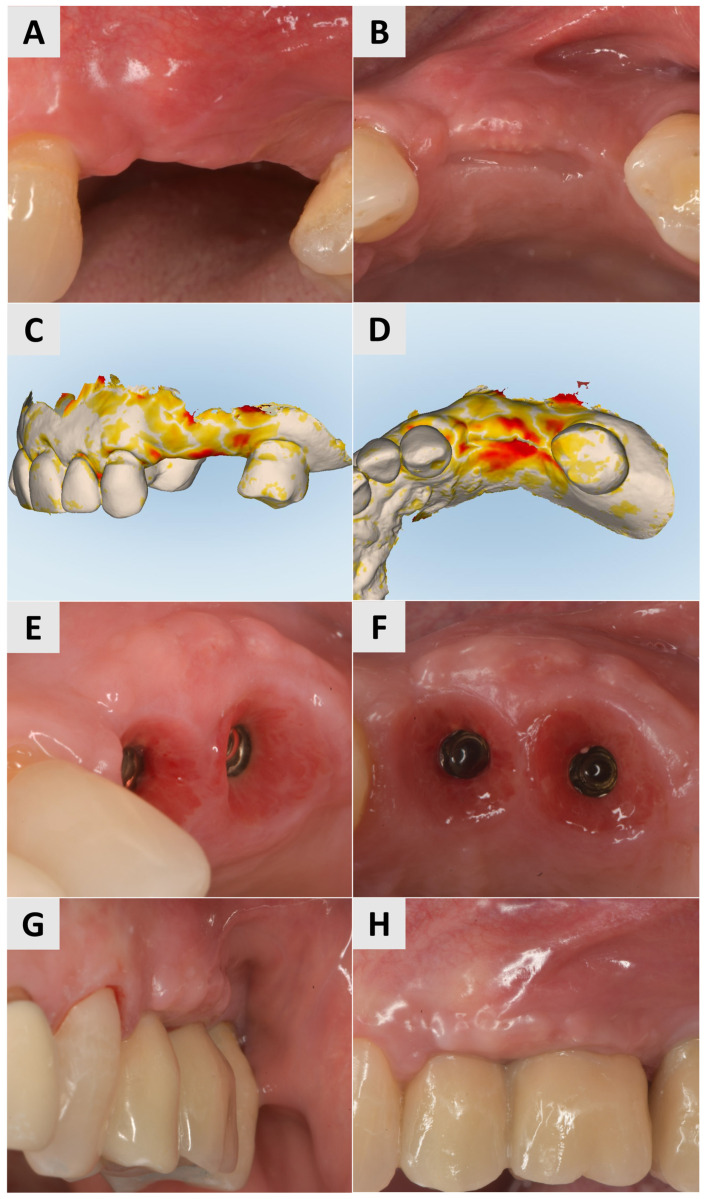
Follow-up for Case 3 at 3-7 months after surgery. (**A**,**B**) Improved harmonization of pink esthetics 3 months after surgery. (**C**,**D**) STL imaging 3 months after surgery, showing successful soft tissue augmentation. **C**) Vertical soft tissue gain of >0.75 mm. (**D**) Horizontal soft tissue gain of >0.75 mm. (**E**,**F**) Implant site 6 months after soft tissue augmentation, presenting with suitable gingival contour and mucosal keratinization. (**G**,**H**) Implant site after crown placement 7 months post intervention.

Three months after the procedure, there was a visible improvement in the keratinization and soft tissue thickness, which contributed to better pink esthetics (Figure 7A,B). STL imaging was performed to assess the 3D soft tissue reconstruction, which showed a vertical increase in soft tissue of more than 0.75 mm and a horizontal increase of more than 1 mm, indicating successful augmentation (Figure 7C,D). At 6 months after surgery, the site showed a suitable gingival contour and the improved keratinization of the mucosa, providing optimal support for the prosthetic restoration (Figure 7E,F). After 7 months, definitive crowns were placed and the implant sites showed a stable tissue volume, esthetic integration and functional readiness (Figure 7G,H).

No complications were encountered in any of the three clinical cases reported. All sites demonstrated stable soft tissue healing with no signs of infection, dehiscence or adverse reactions.

In all cases, re-entry for prosthetic rehabilitation—including implant opening and crown placement—was performed approximately 3 to 6 months after soft tissue augmentation, depending on the individual healing progress and stability of the soft tissue.

Clinical measurements were taken to quantify the soft tissue augmentation achieved in this case series and are summarized in the tables below.

## 4. Discussion

The peri-implant soft tissue quality plays an important role not only in the esthetic outcome but also in the long-term implant survival. Various studies have shown that the presence of sufficiently wide peri-implant keratinized mucosa can lead to less plaque accumulation, less recession and improved soft and hard tissue stability [8,20,38,39,40]. Therefore, a wider zone of keratinized tissue may also be more favourable for the long-term maintenance of dental implants [41]. A thick biotype or surgically increased soft tissue thickness may also reduce the risk of recession in immediate implant placement [42].

Further studies have highlighted the significance of ensuring optimal soft tissue conditions around dental implants to ensure greater marginal bone stability compared to that of sites with minimal keratinized tissue and a minimal mucosal thickness [43]. For example, Linkevicius et al. [38] demonstrated that implants placed at sites with thin soft tissue had significantly larger bone loss compared to implants placed at sites with thick soft tissue. It was also found that the vertical soft tissue thickness plays an important role in the etiology of early crestal bone loss. Therefore, it is just as important to concentrate on the soft tissue outcome as on the hard tissue during regenerative treatments.

To attain a clinically successful outcome for soft tissue grafts, it is imperative that the graft remains completely immobile, as any micromovements can lead to the failure of the graft [11]. Compromised healing and poor vascularization are caused by improper integration with the underlying wound bed and may also influence the shrinkage of the soft tissue graft [44]. For FGGs it is therefore important to quickly stabilize the graft to maintain the nutritional uptake into the graft and reduce shrinkage [45].

Soft tissue grafts are typically secured using sutures; however, these have the disadvantage of compressing the graft and failing to ensure a stable position without micromovements. Additionally, placing too many sutures can hinder the plasmatic circulation of the recipient bed, leading to the creation of micro-separations and compromised graft nutrition [46]. Therefore, fixation screws offer an alternative as a fast and secure method when inserting a soft tissue graft.

A split mouth report by Shi et al. had a patient receive a vestibuloplasty with an FGG affixed with sutures on one side and an acellular dermal matrix (ADM) affixed with tacks on the other side [47]. The outcome showed a significant gain in keratinized tissue at the ADM site when compared to the FGG site. This case report therefore suggests that the use of tacks and an ADM provides faster healing and reduced postoperative morbidity than suturing and using FGGs.

A clinical case series investigated the shrinkage of FGGs secured with titanium tacks up to 6 months postoperatively [23]. It determined that the method provided an ideal fixation result for the graft and assisted in speeding up surgical times. The technique provided good results; however, there was no statistical superiority found when it was compared to other techniques.

Puisys et al. used a PADM in vertical soft tissue augmentation and achieved a mean increase in the soft tissue thickness of 1.8 ± 0.13 mm. In addition, histological evaluation showed a similar number of vessels within the implantation beds compared to the surrounding connective tissue, with no statistical difference in the number of vessels and vascularization [14], highlighting the efficacy of using a PADM for soft tissue augmentation.

In another study, buccal soft tissue augmentation with single implants in an esthetic region performed with a PADM in combination with a CAF resulted in an average increase of 1.2 ± 0.18 mm 1 year after the final restoration [13].

In our cases, the combination of mucoderm^®^ and the magnesium fixation system (NOVAMag^®^ fixation screws) led to measurable and consistent results. As shown in Table 2, the vertical soft tissue measurements ranged from 0.75 mm to 1.05 mm and the horizontal measurements ranged from 0.85 mm to 1.10 mm, with a mean vertical value of 0.86 ± 0.16 mm and a mean horizontal value of 1.00 ± 0.13 mm.

In addition, the detailed clinical parameters in Table 3 show that the thickness of the soft tissue increased from a baseline of 1.0–1.2 mm to 1.9–2.1 mm, while the width of the keratinized mucosa improved by an average of 1.0 mm. These short-term improvements observed after 3 months reflect favourable biological behaviour and support the clinical predictability of this minimally invasive soft tissue augmentation technique. The use of fixation screws instead of sutures stabilizes the membrane and prevents it from tearing and deforming [48], since a stable, immobile membrane is essential for maintaining a good width of the keratinized mucosa [15]. Magnesium fixation screws are degraded on contact with body fluids through a controlled corrosion process that leads to the release of magnesium ions (Mg^2+^), the formation of magnesium hydroxide (Mg(OH)_2_) and the release of hydrogen gas [49]. These degradation products are biocompatible and are either metabolized by the body or excreted without adverse effects. The fixation system is designed to be completely absorbed within about 12 months. Remarkably, magnesium screws retain their biocompatibility even with oral exposure and have not been associated with clinically significant side effects [31,35,50,51,52].Although magnesium screws are resorbable, they have been shown to stabilize the membrane during the critical healing phase [35]. Screws are also a fast method for securing the PADM and, in the author’s experience, faster than the use of sutures.

When suturing the PADM, it is stitched to the periosteum to ensure the supply of vital nutrients and cells whilst maintaining the position of the graft. This can present a challenge for soft tissue grafting at the time of implant placement, which will often require the use of a full-thickness flap. In this situation, the PADM cannot be stabilized by the periosteum and the soft tissue grafting would need to be performed in a second stage of surgery, usually at the point of re-entry. As the use of a fixation screw does not require the involvement of the periosteum, the soft tissue can be augmented in the same procedure as the implant placement.

The combination of PADMs and magnesium fixation screws showed a horizontal and vertical increase in soft tissue after 3 months in all three cases. These clinical results are consistent with the literature, which indicates that a porcine acellular dermal matrix fully integrates within 4–8 weeks, with revascularization beginning as early as 2–4 weeks after insertion [19,20]. The combination of PADMs and magnesium screws promotes revascularization, as a PADM consists of native, non-cross-linked collagen and is quickly integrated and vascularized without showing signs of inflammation [19]. Angiogenesis is very important as it prevents infection and enables good wound healing [35]. Magnesium releases Mg^2+^ ions during its degradation, which stimulate angiogenesis by activating VEGF and other angiogenic factors. Some animal studies have demonstrated the neovascularization and regeneration of vascularized bone 4 weeks after surgery [53].

Overall, the application of the magnesium fixation screws used to secure the PADM, as demonstrated with these three cases, produced very promising results. The screws were quickly inserted, ensuring the stable retention of the graft and preventing micromovements, allowing the full volume of the soft tissue graft to be maintained, which ultimately resulted in a successful soft tissue increase. Compared to suturing the graft, the use of a fixation screw does not require the use of the periosteum to secure its position and maintain contact with the wound bed. It therefore provides the benefit that soft tissue grafting can be performed in the same treatment procedure as implant placement. Additionally, it bypasses the disadvantages of suturing, such as micromovements or compression, that may otherwise lead to insufficient wound bed integration or vascularization of the graft. To fully evaluate the benefits of using magnesium fixation screws over traditional suturing to secure PADM grafts, future research should focus on direct comparative studies with larger sample sizes to fully evaluate the clinical efficacy, benefits and limitations of this technique.

## 5. Conclusions

In this case study, the use of a magnesium fixation system to secure a porcine acellular dermal matrix (PADM) graft was proposed as a technique for soft tissue augmentation. It was successfully performed in a series of three cases and showed similar soft tissue augmentation values to those described in the literature. PADM grafts have the advantage of lower patient morbidity compared to autogenous soft tissue grafts such as CTGs and FGGs, as they avoid the need for further surgery and the associated complications at the donor site. In addition, the use of fixation screws allows soft tissue grafts to be performed at the same time as implantation, as they do not require the use of the periosteum to stabilize the graft. By maintaining stable contact with the wound bed without causing compression or micromovement, this method of augmentation with fixation screws allows for the optimal integration of the graft and a significant increase in the mucosa thickness and keratinization. These results suggest that the combination of PADM grafts and magnesium fixation screws may represent a reliable and patient-friendly approach to improving peri-implant soft tissue outcomes. 

## Figures and Tables

**Table 1:** Colour-coded soft tissue gain based on STL scan analysis.

Colour	Soft Tissue Gain (mm)	Interpretation
**🟡 Yellow**	0.05–0.20 mm	Minimal tissue change
**🟠 Orange**	0.20–0.40 mm	Mild tissue augmentation
**🔴 Red**	0.40–0.75 mm	Moderate augmentation
**🟥 Dark Red**	>0.75 mm	Significant augmentation

**Table 2:** This table shows the vertical and horizontal increases in soft tissue based on STL imaging 3 months postoperatively. The vertical increases ranged from 0.75 mm to 1.05 mm (mean: 0.86 ± 0.16 mm), and the horizontal increases ranged from 0.85 mm to 1.10 mm (mean: 1.00 ± 0.13 mm).

Case	Vertical Soft Tissue Measurements (mm)	Horizontal Soft Tissue Measurements (mm)
1	1.05	1.10
2	0.80	0.85
3	0.75	1.05
**Mean**	**0.86**	**1**
**SD**	**0.16**	**0.13**

**Table 3:** This table shows the detailed clinical parameters of the soft tissue before and after augmentation. The mucosal thickness increased in all the cases from a baseline value of 1.0–1.2 mm to 1.9–2.1 mm, while the increase in the keratinized mucosa was between 0.8 mm and 1.2 mm (mean: 1.0 mm). These results confirm consistent and stable volumetric and qualitative improvements in the peri-implant soft tissue in all three cases.

Case	Initial Soft Tissue Thickness (mm)	Final Soft Tissue Thickness (mm)	Keratinized Soft Tissue Thickness Measurements (mm)
Case 1	1.2	2.0	1.0
Case 2	1.0	1.9	0.8
Case 3	1.1	2.1	1.2

## Data Availability

The data is contained within the article.

## Abbreviations

CTG	connective tissue graft
FGG	free gingival graft
PADM	porcine acellular dermal matrix
ADM	acellular dermal matrix
